# Supplementary material for: An Activatable T1-Weighted MR Contrast Agent: A Noninvasive Tool for Tracking the Vicinal Thiol Motif of Thioredoxin in Live Cells
Source: Molecules. 2021 Apr 1;26(7):2018. doi: 10.3390/molecules26072018 (PMC8037249; doi:10.3390/molecules26072018)
Supplement: Supplementary file 1 [file molecules-26-02018-s001.pdf]

Supporting Information

An activatable T_1-weighted MR contrast agent: Noninvasive tool for tracking the vicinal thiol motif of thioredoxin in live cells

Jongeun Kang,[1,2,†] Eunha Hwang,[3,†] Hyunseung Lee,[1,†] Mi Young Cho,[1] Sanu Karan,[1,2] Hak Nam Kim,[3] Jong Seung Kim,[4] Jonathan L. Sessler,[5,*] Sankarprasad Bhuniya,[1,6,*] and Kwan Soo Hong[1,2,*]

[1]Research Center for Bioconvergence Analysis, Korea Basic Science Institute, Cheongju 28119, Korea

[2]Graduate School of Analytical Science and Technology, Chungnam National University, Daejeon 34134, Republic of Korea

[3]Center for Research Equipment, Korea Basic Science Institute, Cheongju 28119, Korea

[4]Department of Chemistry, Korea University, Seoul 02841, Korea

[5]Department of Chemistry, University of Texas at Austin, Austin, Texas 78712-1224, United States

[6]Centre for Interdisciplinary sciences, JIS Institute of Advanced Studies and Research, JIS University, Kolkata 700091, India

Table of Contents

Figure S1 and S2. ^{1}H and ^{13}C NMR spectra of 1 in $CDCl_3$.. S3

Figure S3. MS spectrum of 1 .... S4

Figure S4 and S5. ^{1}H and ^{13}C NMR spectra of 2 in DMSO-d_6 .... S4

Figure S6. MS spectrum of 2 .... S5

Figure S7 and S8. ^{1}H and ^{13}C NMR spectra of 3 in $CDCl_3$.. S6

Figure S9. MS spectrum of 3 .... S7

Figure S10 and S11. ^{1}H and ^{13}C NMR spectra of 4 in DMSO-d_6 .... S7

Figure S12. MS spectrum of 4 .... S8

Figure S13. MS spectrum of CA1... S9

Figure S14. HPLC spectrum of CA1………………………………………………………….S9

Figure S15. MS spectrum of CA2... S10

Figure S16. Cell viability of MCF-7 cells with T_1-weighted contrast agent CA1........................... S10

Figure S17. MRI of MCF-7 cells incubated with CA1 and CA2 ……………………………….S11

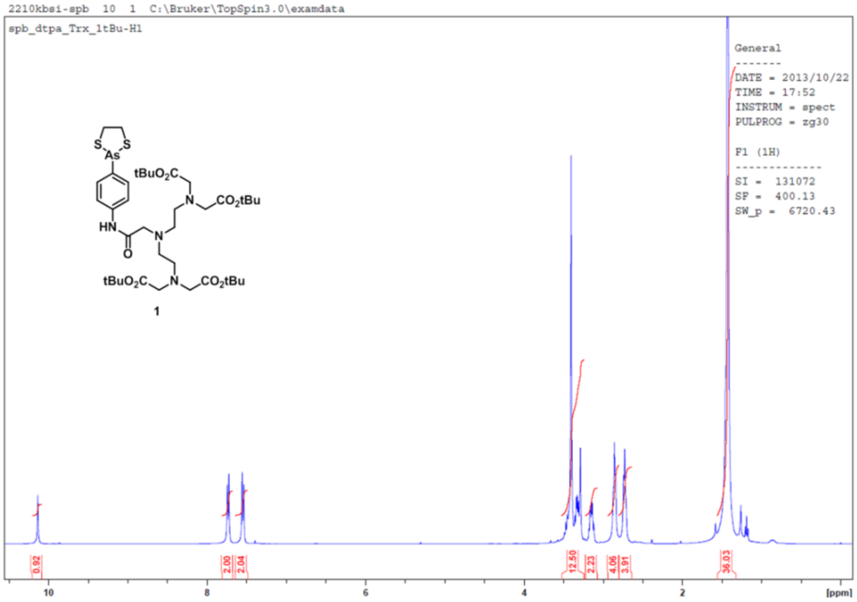

Figure S1. ^{1}H NMR spectrum of **1** in $CDCl_3$.

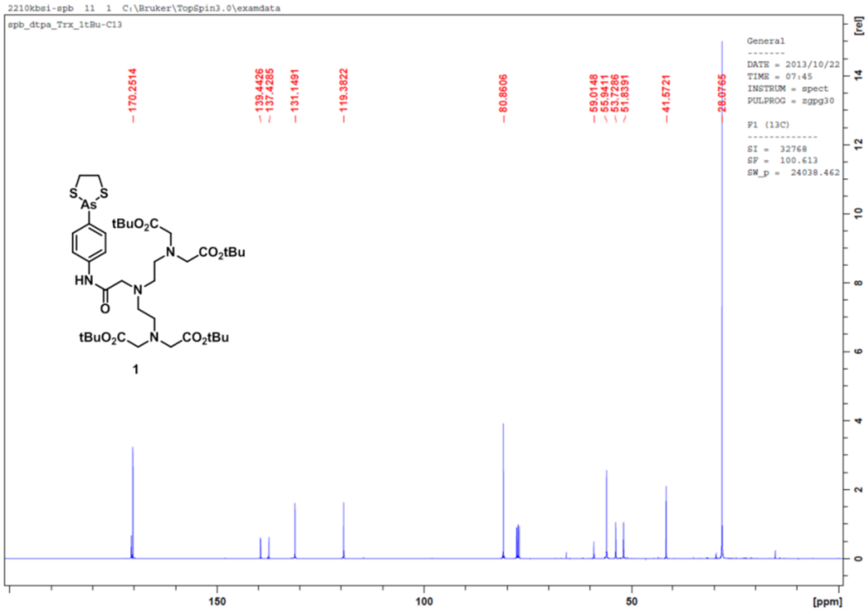

Figure S2. ^{13}C NMR spectrum of **1** in $CDCl_3$.

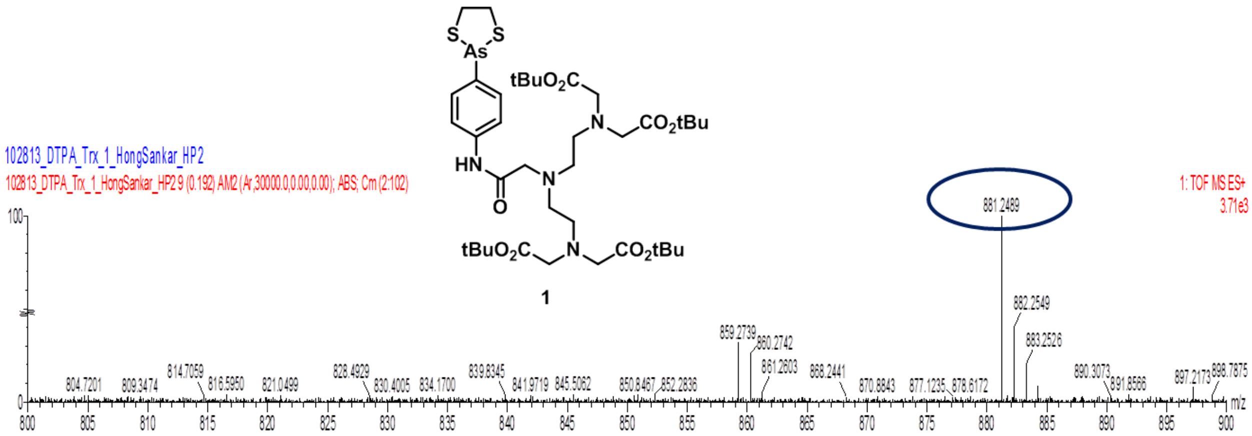

Figure S3. MS spectrum of **1**.

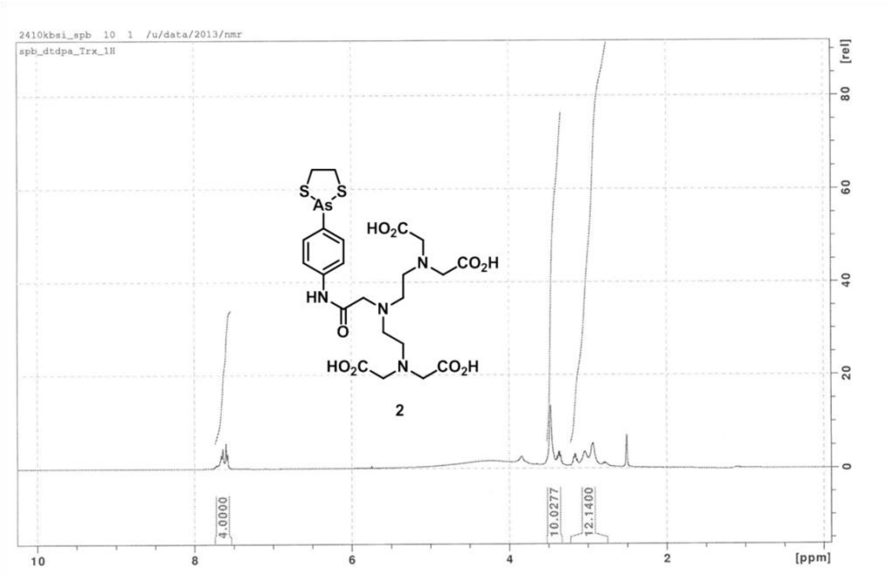

Figure S4. ^{1}H NMR spectrum of **2** in DMSO-d_6.

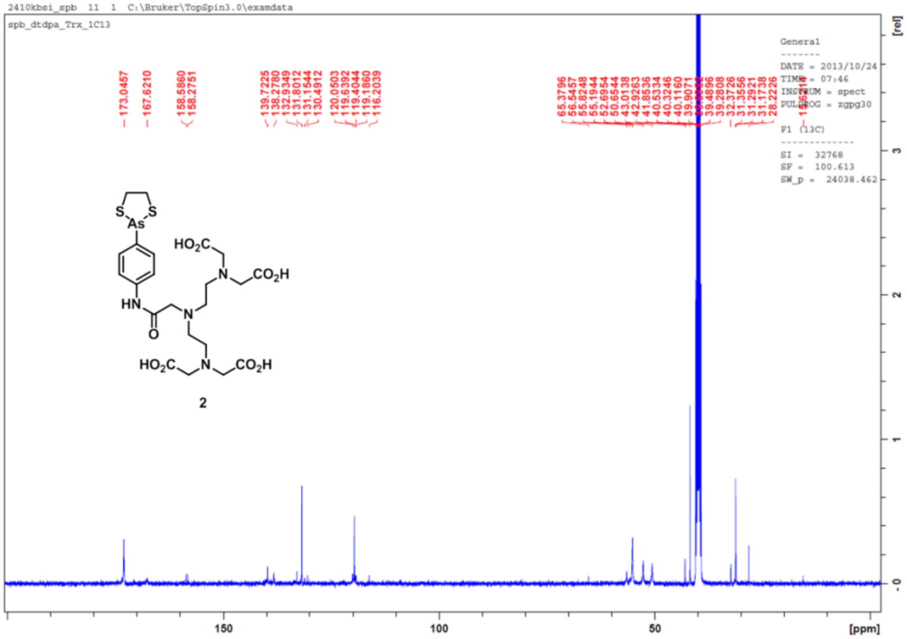

Figure S5. ^{13}C NMR spectrum of **2** in DMSO-d_6.

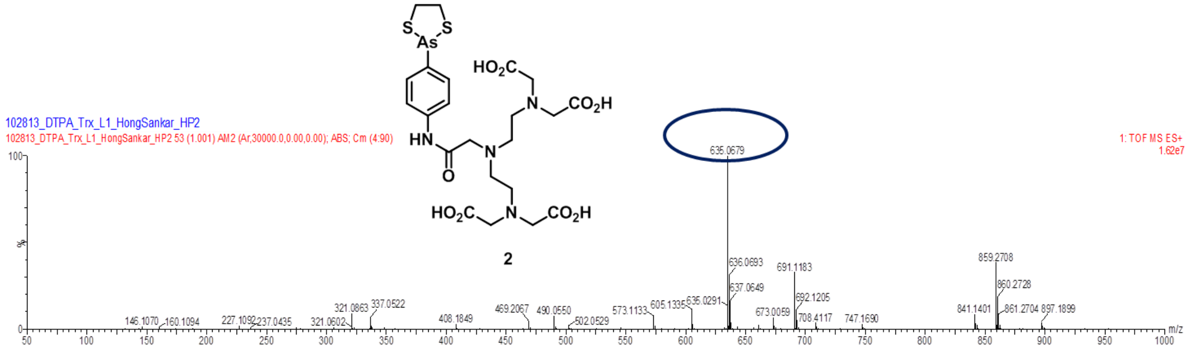

Figure S6. MS spectrum of **2**.

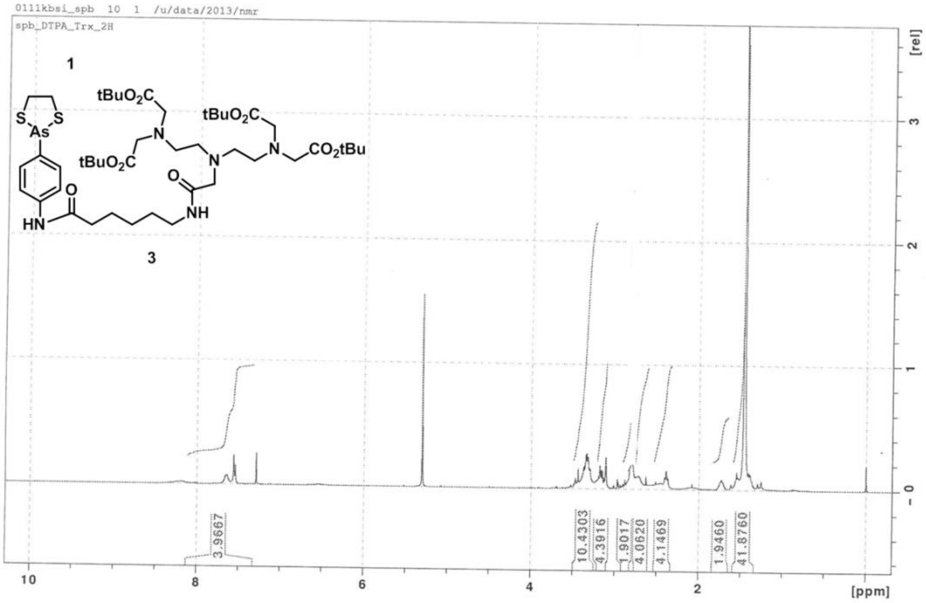

Figure S7. ^{1}H NMR spectrum of **3** in $CDCl_3$.

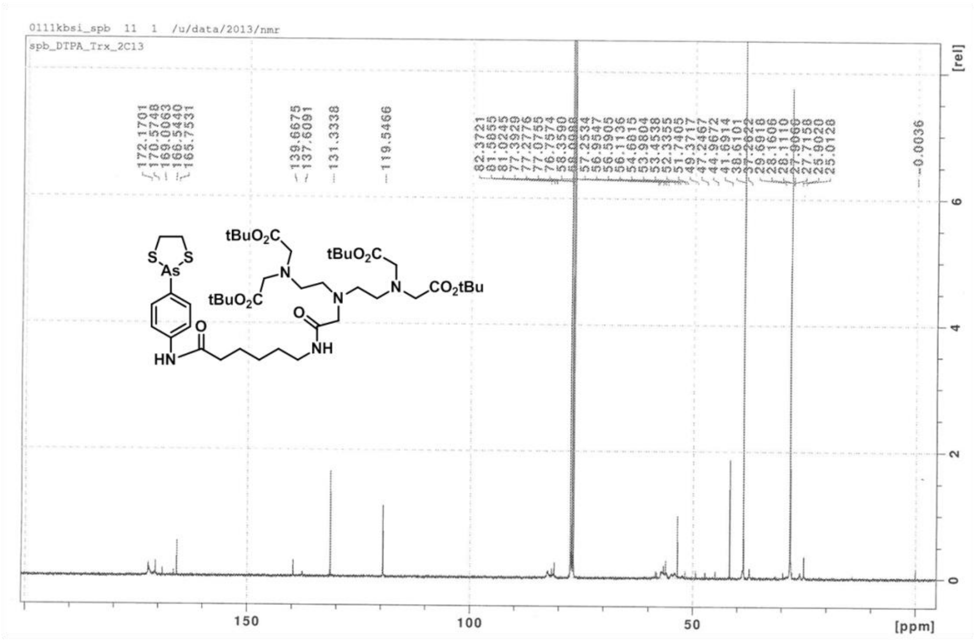

Figure S8. ^{13}C NMR spectrum of **3** in $CDCl_3$.

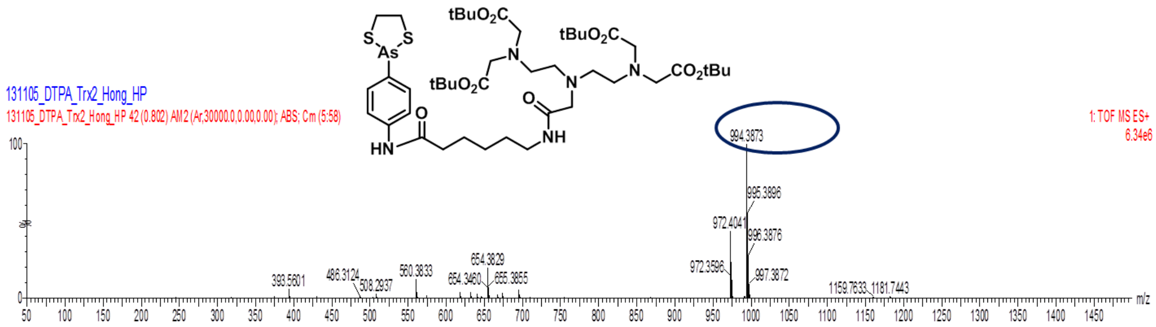

Figure S9. MS spectrum of **3**.

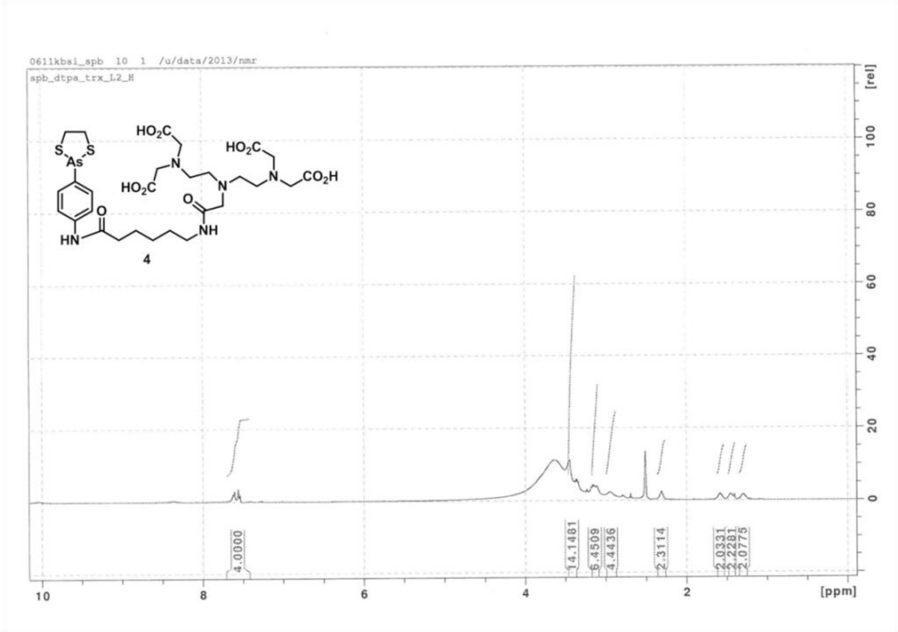

Figure S10. ^{1}H NMR spectrum of **4** in DMSO–d_6.

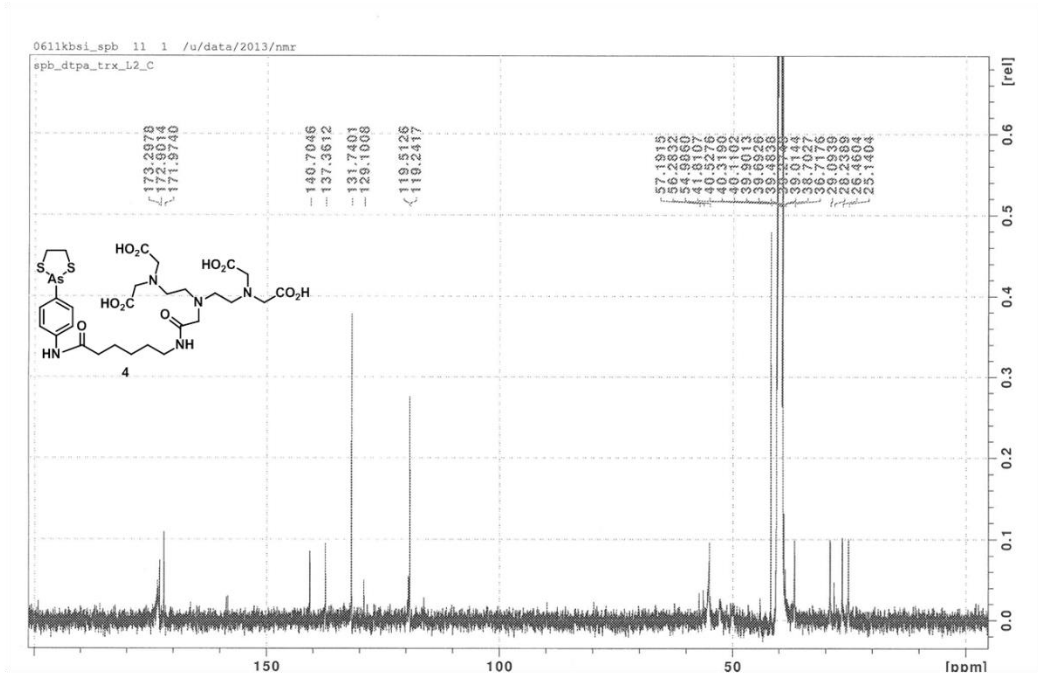

Figure S11. ^{13}C NMR spectrum of **4** in DMSO-d_6.

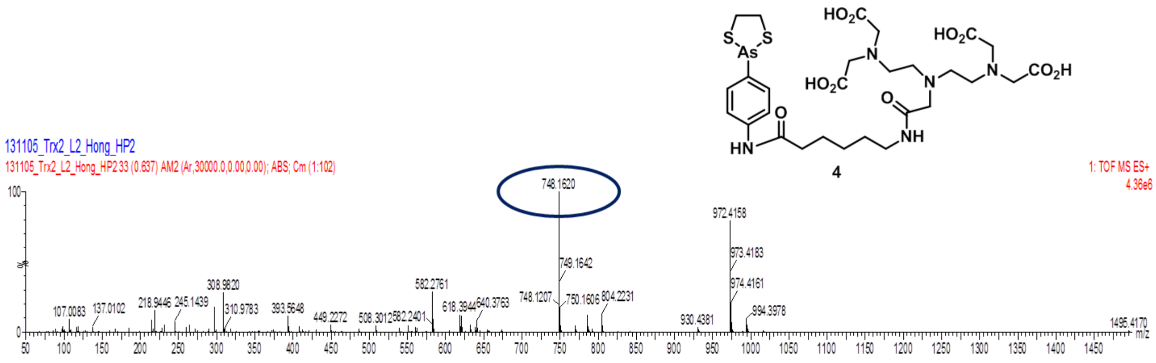

Figure S12. MS-spectrum of **4**.

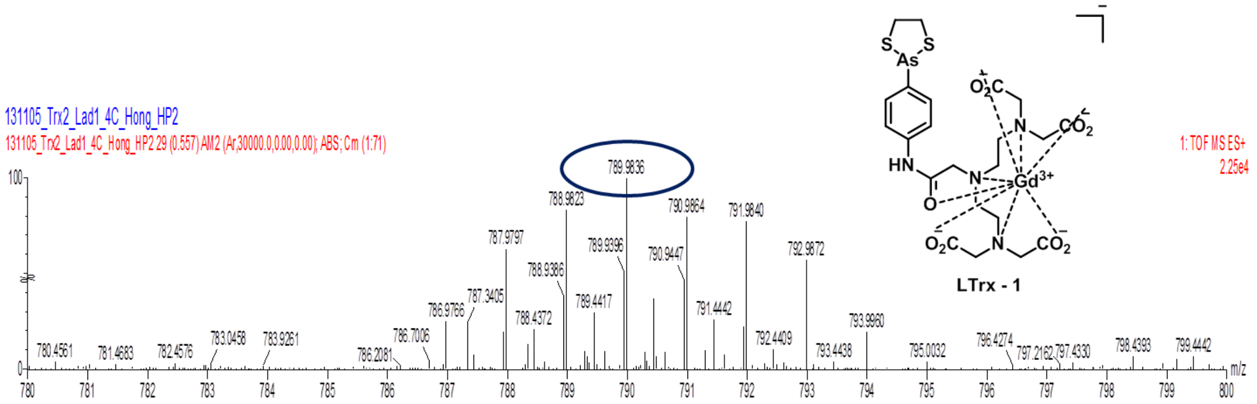

Figure S13. MS spectrum of the Gd^{3+} complex, **CA1**.

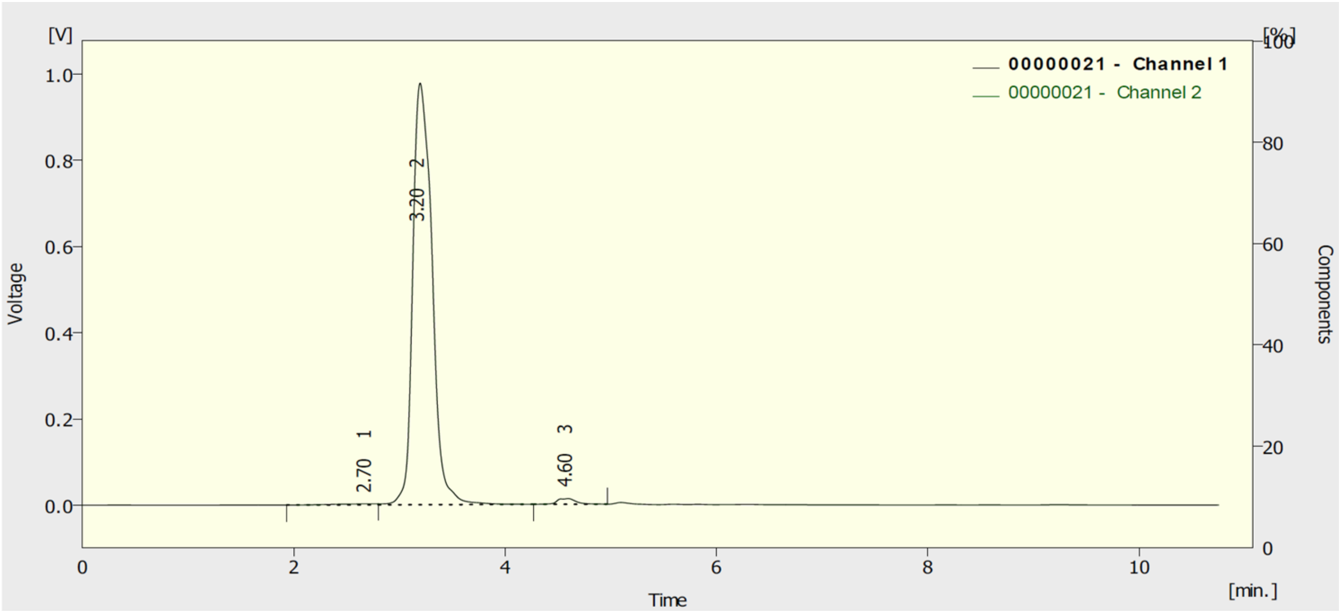

	Reten. Time [min]	Area [mV.s]	Height [mV]	Area [%]	Height [%]	W05 [min]	Compound Name
1	2.697	65.747	2.402	0.5	0.2	0.43	
2	3.197	12424.976	977.861	98.2	98.4	0.20	
3	4.597	166.349	13.314	1.3	1.3	0.19	
	Total	12657.072	993.578	100.0	100.0		

Figure S14. HPLC (high performance liquid chromatography) spectrum of the Gd^{3+} complex, **CA1**.

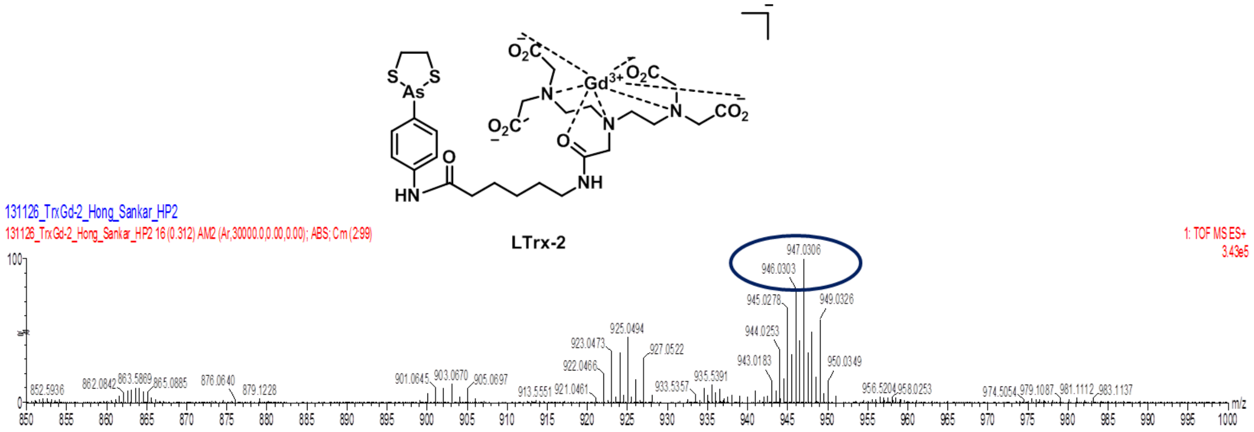

Figure S15. MS spectrum of the Gd^{3+} complex, **CA2**.

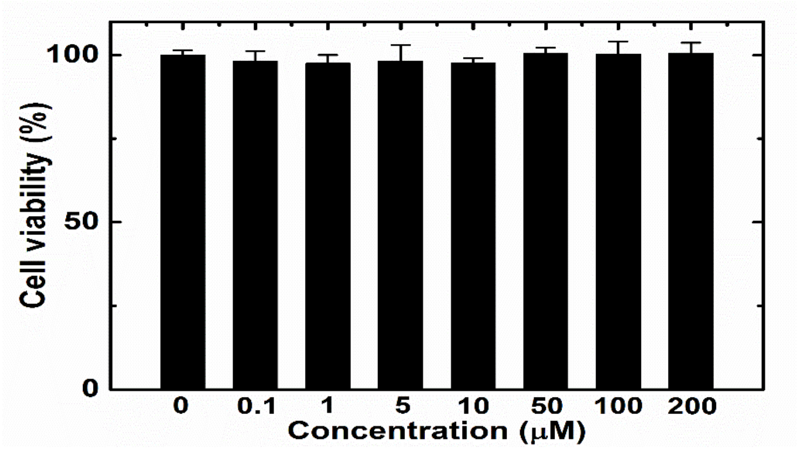

Figure S16. Cell viability of MCF-7 cells after incubation for 24 hours with the T_1 contrast agent **CA1**. The cells were incubated with different doses of **CA1** and viability was measured by means of an MTT assay as described in the main text.

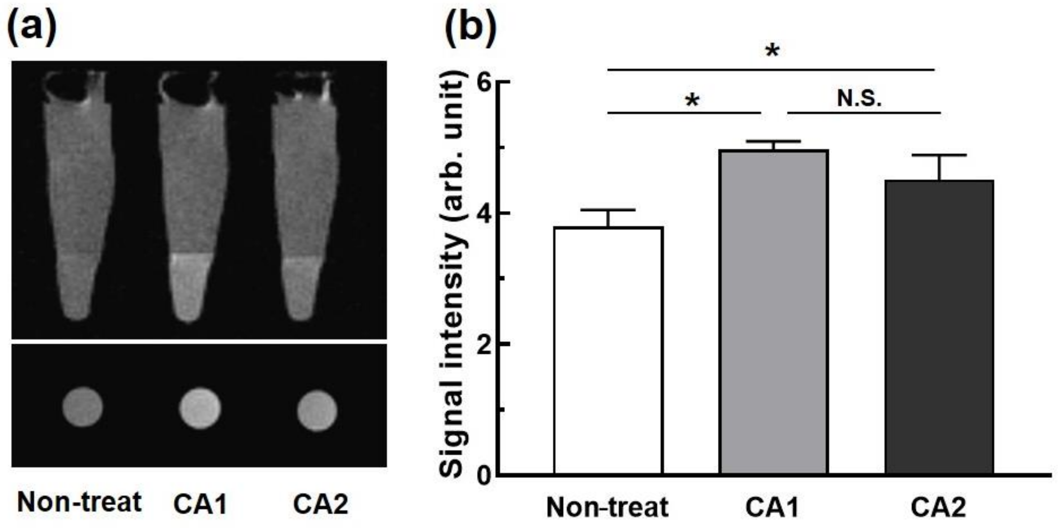

Figure S17. **MRI of MCF-7 cells incubated with CA1 and CA2**. (a) T_1–weighted MR images at 4.7 T at room temperature were obtained in cell phantom samples of MCF-7 cells incubated without (Non-treat) and with **CA1** or **CA2** (100 µM) and (b) corresponding signal intensities measured from region-of-interests of the MR phantom images (study replicated as 2 independent experiments). *, $p < 0.05$ (Student's t-test).
